# Attitudes Toward the Adoption of 2 Artificial Intelligence–Enabled Mental Health Tools Among Prospective Psychotherapists: Cross-sectional Study

**DOI:** 10.2196/46859

**Published:** 2023-07-12

**Authors:** Anne-Kathrin Kleine, Eesha Kokje, Eva Lermer, Susanne Gaube

**Affiliations:** 1 Department of Psychology Ludwig Maximilian University of Munich Munich Germany; 2 Technical University of Applied Sciences Augsburg Augsburg Germany

**Keywords:** artificial intelligence, mental health, clinical decision support systems, Unified Theory of Acceptance and Use of Technology, technology acceptance model

## Abstract

**Background:**

Despite growing efforts to develop user-friendly artificial intelligence (AI) applications for clinical care, their adoption remains limited because of the barriers at individual, organizational, and system levels. There is limited research on the intention to use AI systems in mental health care.

**Objective:**

This study aimed to address this gap by examining the predictors of psychology students’ and early practitioners’ intention to use 2 specific AI-enabled mental health tools based on the Unified Theory of Acceptance and Use of Technology.

**Methods:**

This cross-sectional study included 206 psychology students and psychotherapists in training to examine the predictors of their intention to use 2 AI-enabled mental health care tools. The first tool provides feedback to the psychotherapist on their adherence to motivational interviewing techniques. The second tool uses patient voice samples to derive mood scores that the therapists may use for treatment decisions. Participants were presented with graphic depictions of the tools’ functioning mechanisms before measuring the variables of the extended Unified Theory of Acceptance and Use of Technology. In total, 2 structural equation models (1 for each tool) were specified, which included direct and mediated paths for predicting tool use intentions.

**Results:**

Perceived usefulness and social influence had a positive effect on the intention to use the feedback tool (*P*<.001) and the treatment recommendation tool (perceived usefulness, *P*=.01 and social influence, *P*<.001). However, trust was unrelated to use intentions for both the tools. Moreover, perceived ease of use was unrelated (feedback tool) and even negatively related (treatment recommendation tool) to use intentions when considering all predictors (*P*=.004). In addition, a positive relationship between cognitive technology readiness (*P*=.02) and the intention to use the feedback tool and a negative relationship between AI anxiety and the intention to use the feedback tool (*P*=.001) and the treatment recommendation tool (*P*<.001) were observed.

**Conclusions:**

The results shed light on the general and tool-dependent drivers of AI technology adoption in mental health care. Future research may explore the technological and user group characteristics that influence the adoption of AI-enabled tools in mental health care.

## Introduction

### Background

In spite of the growing efforts to create user-friendly artificial intelligence (AI) applications, their use in clinical care remains limited [1]. Barriers to the adoption of AI-enabled clinical decision support systems (AI-CDSSs) can be found at the individual (eg, end user’s lack of trust in the system), organizational (eg, capacity to innovate), and system (eg, political decisions) levels [2-4]. Often, the adoption of AI-CDSSs fails because system and organizational requirements are not met, and accordingly, tools do not become available to potential end users [5]. The lack of regulatory oversight and standardization of AI-CDSSs can create uncertainty in the field, potentially leading to liability issues at the organizational and system levels [5]. If the system and corporate requirements for implementing a given technology are satisfied, their successful deployment depends on the practitioner’s willingness to use them. However, clinicians may be skeptical about using AI-CDSSs because of concerns regarding the accuracy and reliability of AI-generated decisions. Several frameworks and theories have been developed to systematically study the mechanisms influencing the implementation of technology in practice [5-9]. The 2 most relevant models for individual-level predictors are the Technology Acceptance Model (TAM) [10] and the Unified Theory of Acceptance and Use of Technology (UTAUT) [11]. The TAM aims to explain why a given technology is rejected or accepted by the end user. It proposes that system use is centrally driven by its perceived usefulness and ease of use. Both beliefs are determinants of attitudes toward use, which, in turn, influence use behavior [10]. The UTAUT combines the principles of 8 technology acceptance models, including the TAM. In addition to perceived usefulness (ie, performance expectancy) and perceived ease of use (ie, effort expectancy), it considers social processes (ie, social influence) and demographic variables (ie, age and gender) as predictors of use intention [11]. Accordingly, we focused on the UTAUT as the most holistic use prediction model.

**Figure 1:**
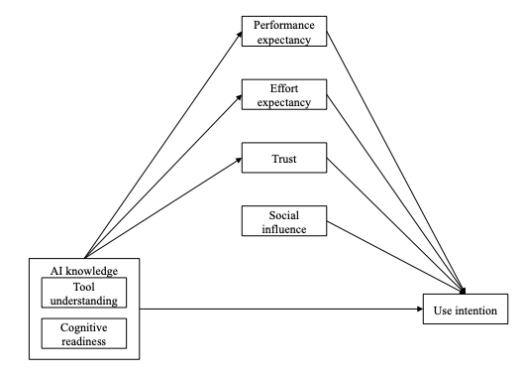
The research model is without control variables. The model is adapted from the preuse part of the model presented in the study by Venkatesh et al [23]. In this study, we extended the original model by adding tool understanding and cognitive technology readiness as predictors of perceived usefulness, perceived ease of use, and trust. AI: artificial intelligence.

Several studies have already demonstrated the applicability of the UTAUT in investigating the implementation of AI-CDSSs [12-17]. However, only 1 study has examined the predictors of the intention to use AI-enabled tools in mental health care [17]. The authors asked psychology students about their general knowledge of and attitudes toward AI systems. The results suggest a link between the perceived social norms, perceived ease of use, perceived usefulness, and perceived knowledge with students’ intention to use AI-enabled tools. However, prospective and current mental health practitioners may have varying levels of skepticism about implementing AI technology for different purposes in their (future) practice. For example, when presented with AI-generated feedback regarding diagnostic or treatment decisions, they may be reluctant to accept AI-based recommendations because of the far-reaching consequences of erroneous predictions or because they feel undermined in their role as therapists. At the same time, they may be open to incorporating AI-generated feedback regarding their interviewing techniques. Although research has begun to examine practitioners’ acceptance of AI-enabled tools in mental health care, there is a lack of specificity in assessing use intention, limiting the utility of these findings in informing practice. This study sought to address this gap by examining the intention to use two specific AI-enabled mental health tools: (1) a psychotherapy feedback tool (FB tool) that analyzes data from therapist-patient conversations and provides performance-specific feedback to the therapist [18-21] and (2) a treatment recommendation tool (TR tool) that uses voice recordings and mood scores to generate recommendations for psychotherapeutic support [22]. The research model is shown in Figure 1.

### The AI-Enabled FB Tool

**Figure 2:**
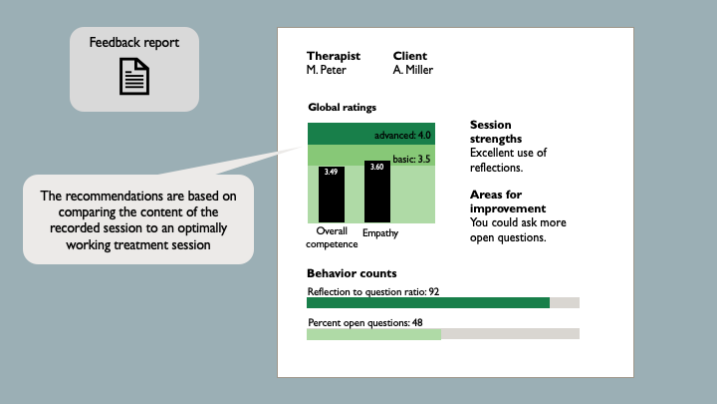
The output slide of the artificial intelligence (AI)–enabled feedback tool showing a visual summary of the AI-generated recommendations regarding the adherence of motivational interviewing principles.

Providing supervision and performance feedback during and after psychotherapy sessions enhances trainees’ and therapists’ skills acquisition and retention [20,23]. However, these processes are labor and cost intensive and thus rarely used in training and clinical practice. Often, feedback is based on trainees’ self-reports and is only available long after the therapy session has concluded [20]. AI technology may help to reduce this problem by providing continuous, immediate, and performance-specific feedback to psychotherapists and trainees. Over the past few years, several AI-enabled FB tools have been developed and are already used in practice [24]. For example, the Therapy Insights Model uses real-time chat messages exchanged between therapists and patients to provide feedback on topics covered in the session and generate recommendations regarding topics that should be addressed in the following session [18]. Counselor Observer Ratings Expert for Motivational Interviewing uses audio recordings of motivational interviewing (MI) sessions to generate feedback on psychotherapists’ adherence to MI principles. The generated feedback focuses on 6 aspects of MI fidelity: empathy, MI spirit, reflection-to-question ratio, percent open questions, percent complex reflections, and percent MI adherence [19]. The tool chosen for this study was developed based on the *Counselor Observer Ratings Expert for Motivational Interviewing*. Participants were presented with information on how speech data recorded during a psychotherapy session were processed and analyzed using machine learning models to generate feedback for psychotherapists regarding their adherence to MI principles and possibilities for improvement, as shown in Figure 2.

### The AI-Enabled TR Tool

**Figure 3:**
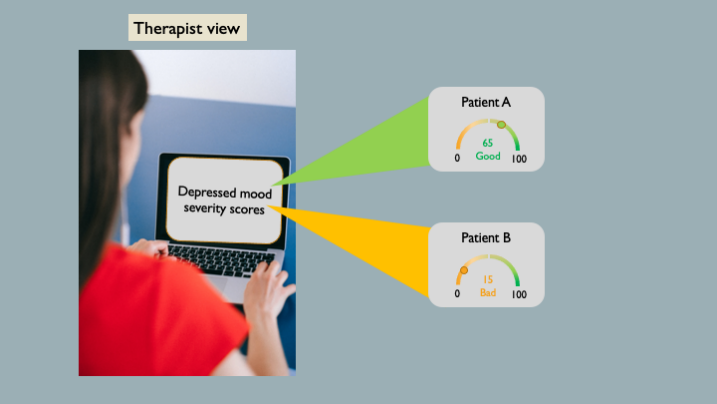
The output slide of the artificial intelligence (AI)–enabled treatment recommendation tool showing a visual summary of the AI-generated mood scores for 2 patients.

Timely psychotherapeutic support may lower the risk of worsening depressive symptoms and suicidality [25]. Multiple studies have demonstrated the effectiveness of AI-enabled emotion analysis in assessing patients’ depressive states and recommending timely intervention, thereby improving mental health care [22,26]. In particular, systems have been developed in recent years to monitor or evaluate the mood of individuals with mental disorders, such as major depressive or bipolar disorder, using speech data [27,28]. These tools usually require patients to record voice samples on their mobile phones, which are analyzed by an automated speech data classifier to assess their current mood [27]. Mental health practitioners can then use this information to decide whether urgent intervention is needed [29]. The TR tool chosen for this study was based on the system developed by *SondeHealth* [30]. Specifically, participants were presented with information on how voice data recorded on a mobile device are processed and analyzed to generate a mood score that may be used for treatment-related decisions, as shown in Figure 3.

### Research Model and Hypotheses

The first goal of this study was to test the applicability of a modified version of the UTAUT in the mental health context to understand the factors that influence the intention to use 2 specific AI-enabled mental health care tools [11,17,31,32]. In line with the UTAUT, we propose tool-specific perceived usefulness (ie, the degree to which an individual believes that using a system will enhance their performance) and perceived ease of use (ie, the degree of ease associated with using the technology) to predict the behavioral intention to use the tools in their future work. The hypotheses for this research have been preregistered through the Open Science Framework [33]. We propose the following hypotheses:

- Hypothesis 1: There is a positive relationship between perceived usefulness and the intention to use the tools in psychotherapy.
- Hypothesis 2: There is a positive relationship between perceived ease of use and the intention to use the tools in psychotherapy.

Unlike experienced psychotherapists, psychology students and psychotherapists in training may be less likely to be influenced by established work habits or procedures, which could impede the adoption of new AI technologies [11]. However, it has been suggested that students are more likely to be affected by their peers and the values and standards of their potential future employers [34]. As a result, we propose that the UTAUT variable, “social influence” (ie, the perception that other significant people think the system should be used), should be considered a predictor of students’ intention to use the tools.

- Hypothesis 3: There is a positive relationship between social influence and the intention to use the tools in psychotherapy.

It has been suggested that trust may be a relevant predictor of the intention to use a technology if the risk associated with it is high [12]. Because of the sensitive nature of the recommendations made by the 2 tools, we hypothesized that trust may be a predictor of students’ intention to use the tools.

- Hypothesis 4: There is a positive relationship between trust in the tools and the intention to use them in psychotherapy.

A lack of understanding of the underlying mechanisms of AI-enabled tools in mental health care has led to skepticism regarding their use [35,36]. In particular, the lack of transparency and explainability of AI-based clinical decision-making has impeded the adoption of such tools in mental health care [35-37]. Building on the new framework for theorizing and evaluating Nonadoption, Abandonment, and Challenges to the Scale-Up, Spread, and Sustainability of Health and Care Technologies [2], we proposed that knowledge regarding technology is a predictor of its perceived value. Consequently, we suggested that students with the knowledge and skills to apply the tools and understand how the recommendations are derived are more likely to perceive them as useful [17,38]. To test this, we extended the UTAUT by including cognitive technology readiness as an indicator of general AI knowledge and understanding of the tool as an indicator of specific AI knowledge as predictors of perceived usefulness, perceived ease of use, and trust. We preregistered 2 research questions to test this relationship:

- Research question 1: Is the positive relationship between cognitive technology readiness and the intention to use the tools mediated through (1) perceived usefulness, (2) perceived ease of use, and (3) trust in the tools?
- Research question 2: Is the positive relationship between understanding of the tools and the intention to use the tools mediated through (1) perceived usefulness, (2) perceived ease of use, and (3) trust in the tools?

## Methods

### Participants

Psychology students and psychotherapists in training were recruited through social media postings, email correspondence with administrative offices of universities, and psychotherapy training centers, as well as through the professional research-focused panel company, Prolific. Data were collected between October 2022 and January 2023, resulting in a total of 362 participants beginning the questionnaire. Of these, 208 provided answers on the behavioral intention to use the tools, resulting in a 42.54% dropout rate. In addition, 2 participants failed at least 2 of the 4 attention check items [39], leaving us with a final sample size of 206.

The final sample consisted of 16% (33/206) of men, 80.1% (165/206) of women, and 3.9% (8/206) of nonbinary individuals. The age of the participants ranged from 18 to 54 (mean 28.10, SD 7.03) years. Data were collected from Germany, the United States, the United Kingdom, and Canada. Most participants studied in Germany (111/206, 53.9%), followed by the United Kingdom (49/206, 23.8%), the United States (32/206, 15.5%), Canada (13/206, 6.3%), and other countries (1/206, 0.5%). Regarding the field of study, most participants stated that their studies focused on clinical psychology (118/206, 57.3%), followed by those studying psychology with no specific focus (50/206, 24.3%) and those who did not provide this information (38/206, 18.4%).

### Procedure

The web-based survey was anonymous and self-administered. All participants provided informed consent before participating. In the web-based survey, we first assessed cognitive technology readiness. Next, participants were presented with slides that explained how recommendations for the AI-enabled *FB tool* and *TR tool* were generated (the material is available from the first author upon request). Before seeing the slides, participants read the following short introduction: “On the following page, you will be presented with a tool that is used to [*FB tool*: provide feedback to psychotherapists about what went well and what could be improved in their sessions; *TR tool*: generate a mood score to rate the severity of patients’ depression. The mood score may be used by psychotherapists to decide which patient to treat first if multiple patients seek treatment and there is limited capacity]. Please read the information carefully and try to understand what the tool does and how it may be used in psychotherapy practice/training. After the presentation, you will be asked a couple of questions about the tool.” After each tool presentation, the UTAUT predictor variables (ie, perceived usefulness, perceived ease of use, social influence, and trust), the understanding of the tool, and the intention to use the respective tool were assessed. Finally, we asked them about their demographic information.

### Ethics Approval

The Institutional Review Board Committee of the University of Regensburg approved the study protocol (22-3096-101).

### Measurement Instruments

#### Independent Variables

We assessed cognitive technology readiness with 5 items of the cognition factor of the medical AI readiness scale [40]. This scale measures terminological knowledge about medical AI applications. In total, 2 items with factor loadings<0.40 [41] that did not relate to a general understanding of AI (ie, “I can define the basic concepts of data science” and “I can define the basic concepts of statistics”) were removed. We retained 3 items related to AI understanding (ie, “I can explain how AI systems are trained,” “I can define the basic concepts and terminology of AI,” and “I can properly analyze the data obtained by AI in healthcare”; α=.77; ω=0.75).

Perceived usefulness, perceived ease of use, and social influence were measured using items adapted from the study by Venkatesh et al [32]. Participants rated their agreement on a 5-point Likert scale ranging from 1=*strongly disagree* to 5=*strongly agree*. Perceived usefulness was assessed using 5 items (eg, “Using the AI tool would enable me to accomplish tasks more quickly”). The reliabilities are α_FB tool_=.86 and ω_FB tool_=0.91 for the first tool and α_TR tool_=.91 and ω_TR tool_=0.93 for the second tool. Perceived ease of use was measured using 4 items (eg, “My interaction with the AI tool will be clear and understandable”; α_FB tool_=.84; ω_FB tool_=0.89; α_TR tool_=.89; ω_TR tool_=0.93). Social influence was measured with 5 items (eg, “In my future job as a psychotherapist, people who are important to me will think that I should use the AI tool”; α_FB tool_=.88; ω_FB tool_=0.94; α_TR tool_=.91; ω_TR tool_=0.95). Trust was measured with 3 items adapted from the study by Venkatesh et al [42] (eg, “The AI tool will provide access to sincere and genuine feedback”; α_FB tool_=.83; ω_FB tool_=0.84; α_TR tool_=.89; ω_TR tool_=0.89). Finally, understanding of the AI-enabled tools was assessed with a single item (“Please rate your understanding of the AI-enabled feedback tool”), with answers ranging from 1=*I don’t understand the tool at all* to 6=*I understand the tool extremely well*.

#### The Behavioral Intention to Use the Tools as the Dependent Variable

The behavioral intention to use the tools was measured on a 5-point Likert scale, ranging from 1=*strongly disagree* to 5=*strongly agree*, with 3 items adapted from the study by Venkatesh et al [32] (eg, “I intend to use the AI tool in my future job as a psychotherapist”; α_FB tool_=.95; ω_FB tool_=0.95; α_TR tool_=.96; ω_TR tool_=0.96).

#### Control Variables

Data privacy concerns and AI anxiety (ie, fears and insecurity regarding AI technology) have repeatedly been identified as negative predictors of the intention to use AI technology [43]. In addition, it has been shown that male participants have more positive attitudes toward AI technologies than female participants [44]. Finally, some evidence exists for the association of AI acceptance with age [45] and country [46]. Accordingly, data privacy and security concerns [47] (α_FB tool_=.84; ω_FB tool_=0.85; α_TR tool_=.89; ω_TR tool_=0.91; eg, “I would be concerned that the AI tool would share my personal information with third-parties”), AI anxiety [32] (α_FB tool_=.78; ω_FB tool_=0.81; α_TR tool_=.76; ω_TR tool_=0.79; eg, “I feel apprehensive about using the AI tool”), gender (0=*man* and 1=*woman and nonbinary*), age, and study country (1=*Germany* and 0=*English-speaking countries*) were included as control variables. One item of the AI anxiety scale and 3 items of the data privacy scales with standardized factor loadings<0.40 were excluded [41].

### Data Analysis

Data were analyzed using R software (version 4.2.2; R Foundation for Statistical Computing) [48]. First, we calculated descriptive statistics, including mean values, SDs, and correlations between study variables for each tool. Second, a confirmatory factor analysis of perceived usefulness, perceived ease of use, social influence, trust, cognitive readiness, specific tool understanding, behavioral intention to use the tool, AI anxiety, and data privacy concerns was conducted using the *lavaan* package [49]. We assumed at least reasonable fit for models with comparative fit index (CFI) and Tucker-Lewis index (TLI) values close to or exceeding 0.90 [50]. Root mean square error of approximation (RMSEA) values <0.08 are considered acceptable [51]. Finally, standardized root mean square residual (SRMR) values up to 0.08 are considered satisfactory [50]. We compared the theoretical measurement model with 3 more parsimonious models (combining cognitive readiness and tool understanding; perceived usefulness and ease of use; and AI anxiety and data privacy concerns) to assess whether the model variables were sufficiently distinct. Third, we conducted structural equation modeling (SEM) using the *lavaan* package [49] to examine the relationships between the predictor variables and the intention to use the tools to answer *hypotheses 1* to *4* and *research questions 1* and *2*. We specified 2 models (1 for each tool) with direct effects and the mediation of the relationship between specific tool understanding, cognitive AI readiness, and the intention to use the tool. We followed the recommendations by Scharf et al [52] to determine whether the regression coefficients should be regularized. Specifically, we applied regularization in case of multicollinearity and associated inflated SEs [52]. The study data and R script will be made available on the web on publication [33].

### Preregistration Statement

The hypotheses were preregistered in the Open Science Framework [33]. Exploratory hypotheses were thus identified.

## Results

Table 1 presents the means, SDs, and correlations. We specified the theoretical model with perceived usefulness, perceived ease of use, social influence, trust, cognitive readiness, specific tool understanding, behavioral intention to use the tool, AI anxiety, and data privacy concerns to load on separate factors. The theoretical model fitted the data adequately (*FB tool*: χ^2^_370_=808.9, *P*<.001; CFI=0.89; TLI=0.87; RMSEA=0.08; SRMR=0.08 and *TR tool*: χ^2^_370_=713.41, *P*<.001; CFI=0.93; TLI=0.92; RMSEA=0.07; SRMR=0.06).

The theoretical model fit the data better than the 3 more parsimonious models (ie, cognitive readiness and specific tool understanding combined; *FB tool*: 𝛥χ^2^_7_=50.37, *P*<.001 and *TR tool*: 𝛥χ^2^_7_=72.68, *P*<.001; perceived usefulness and perceived ease of use combined, *FB tool*: 𝛥χ^2^_8_=257.79, *P*<.001 and *TR tool*: 𝛥χ^2^_1_=435.43, *P*<.001; and AI anxiety and data privacy concerns combined, *FB tool*: 𝛥χ^2^_8_=240.91, *P*<.001 and *TR tool*: 𝛥χ^2^_1_=133.6, *P*<.001). Thus, we concluded that the model variables were sufficiently distinct.

**Figure 4:**
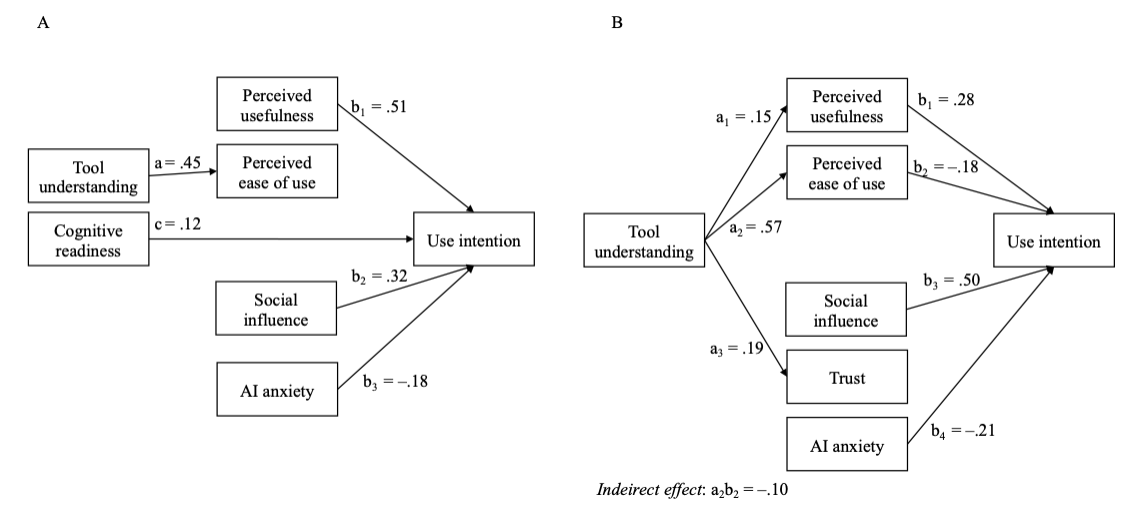
The results of exploratory mediation analysis. Significant paths for the prediction of the intention to use the artificial intelligence (AI)–enabled (A) feedback tool and (B) treatment recommendation tool. Only nonzero paths and indirect effects are displayed.

To test *hypotheses 1* to *4* and *research questions 1* and *2*, we specified 2 SEMs (1 for each tool) with the behavioral intention to use *FB tool* and *TR tool* to be predicted by the respective UTAUT variables (ie, perceived usefulness, perceived ease of use, social influence, and trust); tool understanding; cognitive readiness; and the control variables AI anxiety, data privacy concerns, age, male gender (0=*man* and 1=*woman and nonbinary*), and study country (1=*Germany* and 0=*English-speaking countries*). In addition, we added mediated pathways of the relationship of specific tool understanding and cognitive AI readiness with the intention to use the tools through perceived usefulness, perceived ease of use, and trust in the tool. No inflated SEs were observed, and we proceeded with the interpretation of the SEM without regularization. The results are presented in Tables 2 and 3. Figure 4 shows the significant paths from the SEM path models. As can be seen in Tables 2 and 3 and Figure 4, the relevant paths differ between the 2 models. Perceived usefulness and social influence showed the expected positive relationships with the intention to use both tools, supporting *hypotheses 1* and *3*. However, trust was unrelated to use intention in both models, and perceived ease of use was unrelated to the intention to use the *FB tool* and was negatively related to the intention to use the *TR tool*. Accordingly, we found no support for *hypotheses 2* and *4*. AI anxiety was negatively related to use intentions in both models. Finally, the exploratory mediation analysis results suggest that the relationships of tool understanding and cognitive technology readiness with the intention to use *FB tool* are not mediated through perceived usefulness, perceived ease of use, or trust. There was a negative mediation effect of the relationship between tool understanding and the intention to use the *TR tool* through perceived ease of use, that is, tool understanding was positively related to perceived ease of use, which, in turn, was negatively associated with use intention.

**Table 1:** Means, SDs, and correlations among study variables^a^.

	1	2	3	4	5	6	7	8	9	10	11	12
1. PU^b^	—^c^	0.35	0.74	0.77	0.15	0.73	−0.05	−0.20	0.08	0.05	−0.12	—
2. PE^d^	0.44	—	0.26	0.38	0.54	0.23	−0.26	−0.37	0.05	−0.10	0.01	—
3. SI^e^	0.59	0.40	—	0.71	0.19	0.78	−0.02	−0.25	0.13	0.12	−0.25	—
4. TR^f^	0.68	0.50	0.57	—	0.19	0.72	−0.17	−0.31	0.08	0.04	−0.22	—
5. TU^g^	0.10	0.43	−0.01	0.14	—	0.15	−0.21	−0.19	0.21	−0.13	−0.08	—
6. IU^h^	0.70	0.49	0.67	0.66	0.08	—	−0.11	−0.41	0.11	0.11	−0.23	—
7. PC^i^	−0.07	−0.17	−0.06	−0.24	−0.10	−0.18	—	0.33	−0.11	0.19	−0.03	—
8. ANX^j^	−0.08	−0.31	−0.11	−0.21	−0.22	−0.32	0.31	—	−0.12	−0.11	0.16	—
9. CR^k^	0.07	0.15	0.14	0.09	0.21	0.22	−0.11	−0.19	—	0.01	−0.07	—
10. Age	0.01	−0.03	0.12	−0.06	−0.21	0.02	0.11	−0.03	0.01	—	−0.11	—
11. Gender^l^	−0.08	−0.01	−0.15	−0.11	0.00	−0.12	−0.02	0.11	−0.07	−0.11	—	—
12. Country^m^	−0.10	−0.06	−0.13	0.01	−0.02	−0.03	−0.02	−0.10	−0.02	−0.22	0.21	—
FB tool^n^, mean (SD)	3.2 (0.9)	3.7 (0.8)	2.9 (0.9)	3.4 (0.9)	4.2 (1.0)	2.9 (1.1)	4.2 (1.6)	2.7 (0.9)	2.5 (1.0)	28.1 (7.0)	0.8 (0.4)	0.5 (0.5)
TR tool^o^, mean (SD)	2.8 (1.1)	3.9 (0.8)	2.7 (1.0)	3.0 (1.0)	4.5 (1.1)	2.4 (1.2)	4.0 (1.7)	2.9 (1.0)	2.5 (1.0)	28.1 (7.0)	0.8 (0.4)	5 (0.5)

^a^The lower triangle of the correlation table contains the correlations for the *FBtool*, and the upper triangle contains the correlations for the *TR tool*. All correlations≥|0.14| are significant at *P*<.05.

^b^PU: perceived usefulness.

^c^Not applicable.

^d^PE: perceived ease of use.

^e^SI: social influence.

^f^TR: trust in the tool.

^g^TU: tool understanding.

^h^IU: intention to use the tool.

^i^PC: privacy concerns.

^j^ANX: artificial intelligence anxiety.

^k^CR: cognitive technology readiness.

^l^Code: 0=man and 1=woman and nonbinary.

^m^Code: 1=Germany and 0=English-speaking country.

^n^FB tool: feedback tool.

^o^TR tool: treatment recommendation tool.

**Table 2:** Structural equation modeling results predicting the intention to use the feedback tool (n=206).

Effect		Feedback tool		
		B (SE)	β (95% CI)	*P* value
**Direct effects (DV^a^=IU^b^)**				
	PU^c^	0.63 (0.11)	.51 (.30 to .72)	<.001
	PE^d^	0.06 (0.06)	.03 (−.09 to .15)	.59
	SI^e^	0.37 (0.07)	.32 (.19 to .46)	<.001
	TR^f^	0.06 (0.12)	.04 (−.19 to .27)	.72
	CR^g^	0.12 (0.05)	.12 (.02 to .22)	.02
	TU^h^	−0.07 (0.05)	−.07 (−.18 to .03)	.16
	PC^i^	−0.03 (0.05)	−.04 (−.13 to .06)	.42
	ANX^j^	−0.18 (0.06)	−.18 (−.29 to −.07)	.001
	Age	0.00 (0.04)	−.01 (−.10 to .07)	.74
	Gender^k^	−0.08 (0.04)	−.03 (−.12 to .05)	.48
	Country^l^	0.04 (0.04)	.02 (−.07 to .11)	.66
**Direct effects (DVs=PU, PE, and TR)**				
	TU**→**PU	0.09 (0.08)	.12 (−.03 to .27)	.13
	CR**→**PU	0.04 (0.08)	.04 (−.12 to .20)	.60
	TU**→**PE	0.24 (0.06)	.45 (.32 to .57)	<.001
	CR**→**PE	0.02 (0.07)	.04 (−.11 to .18)	.62
	TU**→**TR	0.09 (0.08)	.13 (−.02 to .28)	.09
	CR**→**TR	0.07 (0.08)	.10 (−.06 to .26)	.22
**Indirect effects**				
	TU**→**PU**→**IU	0.06 (0.04)	.06 (−.02 to .14)	.16
	TU**→**PE**→**IU	0.01 (0.03)	.01 (−.04 to .07)	.59
	TU**→**TR**→**IU	0.01 (0.02)	.01 (−.02 to .04)	.73
	CR**→**PU**→**IU	0.02 (0.04)	.02 (−.06 to .10)	.60
	CR**→**PE**→**IU	0.00 (0.00)	.00 (−.01 to .01)	.71
	CR**→**TR**→**IU	0.00 (0.01)	.00 (−.02 to .03)	.73

^a^DV: dependent variable.

^b^IU: intention to use the tool.

^c^PU: perceived usefulness.

^d^PE: perceived ease of use.

^e^SI: social influence.

^f^TR: trust in the tool.

^g^CR: cognitive technology readiness.

^h^TU: tool understanding.

^i^PC: privacy concerns.

^j^ANX: artificial intelligence anxiety.

^k^Code: 0=man and 1=woman and nonbinary.

^l^Code: 1=Germany and 0=English-speaking country.

**Table 3:** Structural equation modeling results predicting the intention to use the treatment recommendation tool.

Effect			Treatment recommendation tool		

		B (SE)		β (95% CI)	*P* value
**Direct effects (DV^a^=IU^b^)**					
	PU^c^	0.31 (0.11)		.28 (.06 to .50)	.01
	PE^d^	−0.29 (0.06)		−.18 (−.30 to −.06)	.004
	SI^e^	0.56 (0.08)		.50 (.34 to .65)	<.001
	TR^f^	0.23 (0.11)		.17 (−.04 to .37)	.12
	CR^g^	−0.01 (0.04)		.00 (−.09 to .08)	.91
	TU^h^	0.02 (0.05)		.02 (−.07 to .12)	.65
	PC^i^	−0.01 (0.05)		−.01 (−.10 to .08)	.81
	ANX^j^	−0.25 (0.06)		−.21 (−.33 to −.10)	<.001
	Age	0.00 (0.04)		−.02 (−.10 to .06)	.64
	Gender^k^	−0.04 (0.04)		−.01 (−.09 to .07)	.74
	Country^l^	−0.08 (0.04)		−.03 (−.11 to .04)	.40
**Direct effects (DVs=PU, PE, and TR)**					
	TU**→**PU	0.15 (0.07)		.15 (.01 to .29)	.04
	CR**→**PU	0.04 (0.08)		.04 (−.12 to .19)	.64
	TU**→**PE	0.40 (0.05)		.57 (.47 to .68)	<.001
	CR**→**PE	−0.06 (0.07)		−.07 (−.20 to .06)	.30
	TU**→**TR	0.15 (0.07)		.19 (.04 to .33)	.01
	CR**→**TR	0.06 (0.08)		.06 (−.10 to .22)	.44
**Indirect effects**					
	TU**→**PU**→**IU	0.05 (0.03)		.04 (−.01 to .09)	.12
	TU**→**PE**→**IU	−0.11 (0.04)		−.10 (−.18 to −.03)	.01
	TU**→**TR**→**IU	0.03 (0.02)		.03 (−.01 to .08)	.19
	CR**→**PU**→**IU	0.01 (0.02)		.01 (−.03 to .05)	.64
	CR**→**PE**→**IU	0.02 (0.01)		.01 (−.01 to .04)	.34
	CR**→**TR**→**IU	0.01 (0.01)		.01 (−.02 to .04)	.49

^a^DV: dependent variable.

^b^IU: intention to use the tool.

^c^PU: perceived usefulness.

^d^PE: perceived ease of use.

^e^SI: social influence.

^f^TR: trust in the tool.

^g^CR: cognitive technology readiness.

^h^TU: tool understanding.

^i^PC: privacy concerns.

^j^ANX: artificial intelligence anxiety.

^k^Code: 0=man and 1=woman and nonbinary.

^l^Code: 1=Germany and 0=English-speaking country.

## Discussion

### Principal Findings

In recent years, there has been a rapid growth in the development of AI-enabled mental health care tools. To investigate the implementation challenges and potential user needs, in this study, we examined the intention to use 2 AI-enabled mental health care tools among psychology students and psychotherapists in training. The first tool provides feedback to the psychotherapist on their adherence to MI techniques by analyzing data collected during psychotherapy sessions. The second tool uses patient voice samples to derive mood scores that the therapists may use for treatment decisions. An extended UTAUT model was used to analyze the results, which showed that perceived usefulness and social influence had a positive effect on the intention to use both tools. However, trust was unrelated to the intention to use both tools, and perceived ease of use was unrelated (FB tool) and even negatively related (TR tool) to the intention to use when considering all predictors in 1 model.

The findings of this study are partly in line with previous research on AI-CDSSs in medicine [13,15]. Fan et al [13] found positive associations between perceived usefulness and trust with use intentions among a sample of health care professionals, and Zhai et al [15] reported positive relationships between perceived usefulness and social influence with the intention to use AI-assisted contouring technology among radiation oncologists. Furthermore, Tran et al [16] identified social influence as the only significant predictor of the intention to use AI-CDSSs among undergraduate medical students. Gado et al [17] found support for the direct effects of perceived usefulness, AI knowledge, and perceived social norms on the intention to use AI as well as indirect effects of perceived ease of use on use intention via positive attitudes toward AI in a sample of psychology students. This consistent link between social influence and AI use intentions found in studies using student samples may be explained by the greater susceptibility of students to influence of peers and prospective employers [53]. As students have yet to develop a professional identity that shapes their work-related decisions, they may be more likely to align their decisions with the perceived expectations of influential others [54].

The assessment of symptom severity often involves complex interactions with the patient and reflections on psychotherapeutic elements, which may make participants skeptical of a device that is perceived as being easy to use. One explanation for the null and negative relationships between perceived ease of use and use intentions for AI-generated recommendations in the mental health field may be the high stakes of accepting the tool’s advice. This interpretation might be supported by a study predicting intentions to learn about AI applications among medical staff [55], which found that perceived ease of use was the strongest predictor of the intention to learn how to use AI-enabled tools in health care. Combined with the results of this study, it may be assumed that ease of use positively predicts interactions with AI-generated advice that aligns with the user’s level of competency and professionalism. That is, ease of use may positively predict learning intentions but maybe not the intention to use high-stakes mental health tools among students and trainees who have not yet gained profound professional experience. Students’ primary task at university is to learn and acquire skills and knowledge. The ease with which an AI-enabled tool can be applied likely becomes more relevant when the interaction with such tools is required or advantageous for their professional performance. More research is needed to understand the conditions under which perceived ease of use is positively related to AI use intentions among medical and mental health practitioners and to explore the implications of the high stakes associated with AI-generated recommendations.

Trust in the tools was unrelated, whereas AI anxiety was negatively related to the intention to use both the FB and TR tools. One explanation for this finding may be participants’ limited insight into the functioning mechanisms of the tools. A profound assessment of their trust in the tools requires more in-depth knowledge than assessing their AI anxiety. Specifically, whether the AI tool “will provide data in [their] best interest,” “provides access to sincere and genuine feedback,” or “will perform its role of a supportive system very well” [42] may be difficult to assess without having used the tool in practice and, thus, may be less relevant for students’ intention to use the tool. In contrast, AI anxiety represents intuitive, affective reactions, such as feeling apprehensive about the tool or being hesitant to use the tool for fear of making mistakes [32]. As students and psychotherapists in training have limited to no experience interacting with AI-generated feedback, they may base their decision-making on intuitive, emotional reactions better represented by AI anxiety than trust in the tools [56].

By differentiating between specific tool understanding and more general cognitive technology readiness, this study moves beyond previous research that focused on the role of general AI knowledge in predicting general use intention [17]. The mediation analyses revealed that none of the 3 UTAUT variables mediated the relationship between tool understanding and cognitive technology readiness with the intention to use the FB tool. However, there was a positive relationship between cognitive technology readiness and the intention to use the FB tool. This might indicate that general AI understanding may spur use intentions of low-stakes AI-generated advice but not the intention to use AI advice for deriving treatment decisions. In addition, in line with the direct effects, perceived ease of use emerged as a negative mediator between specific tool understanding and the intention to use the TR tool. The results of the exploratory mediation models highlighted the relevance of distinguishing between different AI-enabled tools when assessing the relationship between different forms of AI knowledge and use intentions.

### Limitations and Future Directions

This study has some limitations. First, we collected data at only 1 time point. Although cross-sectional designs are commonly chosen to investigate mechanisms predicted by the UTAUT [13,15], they prevent the assessment of an order of effects. The adoption of AI-generated advice should be studied longitudinally to increase the understanding of use-predicting mechanisms. Second, although studying technology acceptance with deterministic models, such as the UTAUT and TAM, has a long tradition, such studies have recently been criticized for their oversimplicity, which lowers their explanatory power. In this vein, focusing on 2 specific AI-enabled mental health tools may be highlighted as a strength of this study, as it increases the ecological validity of the results. However, future research should seek to integrate organizational and system processes to provide a more profound understanding of the mechanisms that prevent and promote technology adoption. Other frameworks and theories, such as activity theory [57], adaptive structuration theory [58], and the Nonadoption, Abandonment, and Challenges to the Scale-Up, Spread, and Sustainability of Health and Care Technologies framework [2], may serve as theoretical underpinnings of research investigating use in context instead of focusing on individual-centered variables alone [5]. Finally, we focused on psychology students and psychotherapists in training as a potential user group and found discrepancies in our results compared with previous research findings [13,16]. Future research should compare adoption and adoption intentions among multiple (potential) user groups and tools to shed light on tool-dependent and user-dependent predicting mechanisms.

### Conclusions

This study provides insights into the individual implementation challenges of AI-enabled FB and TR tools used in mental health care. The results highlight the relevance of specific UTAUT predictors as general drivers of AI technology adoption in mental health care (ie, perceived usefulness, social influence, and AI anxiety) and emphasize the need to distinguish between different AI technologies with reference to other influencing factors (ie, perceived ease of use, cognitive technology readiness, and tool understanding). Future research should explore the conditions under which perceived ease of use is positively related to AI use intentions among mental health practitioners.

## Abbreviations

AI: artificial intelligence
AI-CDSS: artificial intelligence–enabled clinical decision support system
CFI: comparative fit index
FB tool: feedback tool
MI: motivational interviewing
RMSEA: root mean square error of approximation
SEM: structural equation modeling
SRMR: standardized root mean square residual
TAM: Technology Acceptance Model
TLI: Tucker-Lewis index
TR tool: treatment recommendation tool
UTAUT: Unified Theory of Acceptance and Use of Technology
